# Efficacy and safety of seawater-based nasal irrigation on nasal congestion in infants and toddlers with common cold: a randomized controlled trial

**DOI:** 10.1007/s00405-026-10615-z

**Published:** 2026-09-16

**Authors:** Oliver Chen, Constance Prime, William Hooper, Thomas Vanderzande, Marita Alegre, Anna Rynkiewicz, Anetta Karpińska-Nowak, Annahita Ghassemi

**Affiliations:** 1 Church & Dwight, Princeton, NJ 08540 USA; 2 Sofibel-Church & Dwight, 92300 Levallois-Perret, France; 3 Church & Dwight (U.K.) Ltd, Folkestone, UK; 4 NZOZ Medicus, Os. Sierakowskich 5, 82-400 Sztum, Poland; 5 NZOZ Nowy Chełm, Ul. Tytusa Chałubińskiego 23, 80-807 Gdańsk, Poland

**Keywords:** Flu, Rhinovirus, Pediatric, URTI, Copper, Manganese, Hypertonic, Osmosis, Secondary infection, Stuffy nose, Adjunct therapy, Nasal irrigation, Saline nasal lavage, Symptom relief

## Abstract

**Background:**

Nasal congestion associated with the common cold is a frequent source of distress in infants and toddlers. While hypertonic seawater solutions offer a physiological mechanism for decongestion via osmosis, robust clinical evidence supporting their use as an adjunct to standard care in this specific pediatric population is essential to validate their therapeutic value.

**Objective:**

To evaluate the efficacy and safety of a hypertonic seawater nasal spray enriched with copper and manganese combined with standard of care compared to standard of care alone in alleviating nasal congestion, improving sleep quality, and accelerating symptom recovery in infants and toddlers aged 3–48 months experiencing the common cold without high fever (excluding acute rhinosinusitis).

**Methods:**

In a randomized, open-label, controlled parallel study, 220 participants received standard care (which included ample hydration, at-home rest, and the use of paracetamol as needed) with or without the nasal spray. Outcomes, including symptom severity, recovery rate, sleep quality, secondary infections, and caregiver satisfaction, were assessed via questionnaires over 10 days.

**Results:**

Participants using the nasal spray experienced a significant (*p* < 0.05) reduction in nasal congestion from the evening of Day 0 through Day 5, with immediate symptom relief observed within 5–15 min of application. By Day 10, recovery rates were higher in the nasal spray group (75.3% vs. 58.5%). Improvements in cough and runny nose severity were significant, though minimal effects were noted for other symptoms. Sleep quality and secondary infection rates were comparable between groups. Caregivers expressed high satisfaction, with 93.6% willing to use the product again. No adverse events were linked to the nasal spray.

**Conclusion:**

The hypertonic seawater solution nasal spray is a safe and effective adjunct to standard care, providing rapid relief of nasal congestion and improving cold recovery rates in young children. Trial registered at ClinicalTrials.gov: NCT05365789 (https://clinicaltrials.gov/search?cond=NCT05365789).

**Supplementary Information:**

The online version contains supplementary material available at https://doi.org/10.1007/s00405-026-10615-z.

## Background

Upper respiratory tract infection (URTI) such as the common cold is one of the most prevalent illnesses globally and a leading cause of medical visits and absences from daycare, school, and work [1]. Viral URTI is caused by more than 200 types of viruses including rhinoviruses, coronaviruses, respiratory syncytial virus (RSV), parainfluenza viruses, along with adenoviruses and various unidentified viruses [2]. Because new cold viruses emerge continually, the body cannot develop immunity to all of them, resulting in recurrent infections. Infants and young children are particularly susceptible, with those under 2 experiencing up to 8–10 colds per year, while school-aged children typically have 6–12 [3]. Adolescents and adults, having built immunity to many viruses, average only 2–4 colds annually [3]. Children in group care environments such as daycare experience more frequent colds, transmitted through respiratory secretions and close caregiving contact [4].

Cold symptoms vary by virus and age. In infants and toddlers, common symptoms include runny nose, nasal congestion, sneezing, cough, and mild sore throat, with occasional low-grade fever. While colds are self-limiting, typically lasting about a week, they can extend to 2 weeks. Importantly, mild cold viruses in older children and adults can lead to more severe respiratory symptoms in infants and toddlers, such as croup and swollen glands, making symptom management crucial in this age group [5]. Currently, there is no cure for the common cold, with treatments aimed at alleviating symptoms while the body clears the infection. Over-the-counter (OTC) medications, while effective for older children and adults, are generally unsuitable for those under 3 years old without medical advice [6, 7]. Thus, non-pharmacologic methods such as rest, hydration, humidifiers, nasal suction devices, and saline sprays are preferred methods to provide some comfort for young children.

Nasal irrigation is an established, low-cost approach for managing nasal and sinus symptoms. It involves rinsing the nasal cavity with a saline solution to enhance mucociliary clearance, reduce congestion, and remove pathogens and inflammatory agents. Saline irrigation, however, can be challenging in very young children. In such cases, saline sprays or drops may offer a more practical solution for infants and toddlers with nasal congestion [8]. In addition to the method of application, the choice of irrigation solution is also important. Evidence suggests that hypertonic saline solutions offer a stronger decongestant effect by drawing out excess water from swollen mucous membranes through osmosis than isotonic ones, thereby reducing nasal swelling and improving nasal ciliary function [9]. This study evaluates the efficacy, safety, and tolerability of the Stérimar Blocked Nose Baby nasal spray in children. Designed specifically for infants and toddlers, this Class IIa medical device contains a hypertonic seawater solution enriched with essential trace elements such as manganese and copper. It is preservative-free and intended for use in infants and toddlers, pregnant and breastfeeding women, and individuals who prefer non-pharmacologic options. This study aims to address the need for effective, safe symptom relief for nasal congestion in infants and toddlers with the common cold by evaluating whether the addition of a seawater-based nasal spray to standard care improves outcomes compared to standard care alone.

## Methods

### Study design

This was a randomized, open-label, controlled parallel study whereby participants received standard of care with or without the use of the Stérimar Blocked Nose Baby spray until nasal symptoms were resolved. Cold symptom severity, occurrence of secondary infections, onset of relief, use of concomitant medications, quality of sleep, and common cold status were assessed by questionnaires throughout the intervention period. Ethical approval for this study was obtained from the Bioethics Committee of the Regional Chamber of Physicians (Gdańsk, Poland). This study was conducted per the ethical principles initially outlined in the Declaration of Helsinki (1964) and according to EN ISO standard 14,155:2020 and EU regulation 2017/745 on medical devices and complies with local regulatory requirements. Signed informed consent and authorization for the use of protected health information were provided by each participant’s parents/legal guardians before implementing any protocol-specific procedures. The study was managed by Eurofins Dermscan Poland (Gdańsk, Poland) and was conducted between January 20, 2022, and August 4, 2023, at two clinics in Poland. CONSORT reporting guidelines were used [10]. This trial was registered on ClinicalTrials.gov (NCT05365789).

### Study participants

Participants were otherwise healthy male and female infants and toddlers, 3—48 months old, who experienced at least one cold symptom (e.g., runny nose, nasal crust (dry mucus), thick mucus, sneezing, or coughing) starting within 48 h before enrolment. Additionally, all participants needed to have nasal congestion (blocked/stuffy nose) rated as at least a score of 2 (moderately bothersome) on a 0 to 3-point scale, based on morning evaluation (within an hour of participant awakening). To determine eligibility, body temperature measurements and streptococcal rapid antigen detection tests (RADT) were performed on all participants during the initial screening phase. Exclusion criteria included body temperature greater than 38 °C, presenting secondary infection (e.g., bronchitis, tracheitis, pneumonia), positive results on the streptococcal antigen screening test (RADT), history of allergic rhinitis, congenital or chronic disease that would adversely affect the results of the study (e.g., asthma, pneumonia, laryngotracheobronchitis, sinusitis), immunodeficiency, hypersensitivity or allergy or intolerance to any component of the study product or of the rescue medication (paracetamol or acetaminophen for pain, headache, or fever relieve), active systemic infection or medical condition that may require treatment or therapeutic intervention during the study, use of saline nose drops or nasal sprays or pumps other than the study products, antibiotics, antivirals, intranasal medicines, decongestants, antihistamines, echinacea, combination cold formulas, supplements containing ≥ 10 mg zinc that would influence symptoms scores at enrolment, was in a country with a higher incidence rate of COVID-19 than Poland within 14 days of enrolment, or had contact with any person with COVID-19, currently participating or had participated in another trial within 30 days prior to enrolment, or is currently home-quarantined.

### Study interventions

All participants were recommended standard of care which included ample hydration (offering drinks, possibly warm, to restore water loss), at-home rest, and use of paracetamol as needed and following consultation with the study investigators. Any use of paracetamol was recorded. In addition to standard of care, participants randomized to the active group also used the Stérimar Blocked Nose Baby nasal spray at least twice a day (1–2 sprays per nostril in the morning and evening) and as needed up to a maximum of 6 times per day (i.e., maximum of 12 sprays per nostril per day). For infants between 3 months and 2 years old, caregivers were instructed to apply the spray with the infant in a supine position with the head tilted to the side (not backwards). Meanwhile, for children 2 years and older, the spray was applied with the child in a sitting position with the head tilted to the side. Caregivers were instructed to clean the nozzle of the spray thoroughly with hot water between applications.

Participants used the nasal spray until nasal symptoms resolved, defined as no reported symptoms for 2 days using the “global assessment on common cold status” questionnaire, or up to a maximum of 10 days. The use of the nasal spray was recorded in a daily log. Because the pressurized Bag-On-Valve spray device does not deliver a precise metered dose per actuation, the weight of the device before and after the end of the study was measured to accurately determine the total quantity of product used by each participant during the study.

The Stérimar Blocked Nose Baby (SOFIBEL Laboratoires Fumouze, France, a subsidy of Church & Dwight UK Ltd., England) consists of a hypertonic sea water solution from the bay of Cancale with a higher salt concentration than the human body’s cells for quick decongestion of blocked noses by osmosis effect. The hypertonic seawater solution is rich in minerals salts and marine trace elements and enriched with copper (copper sulfate) and manganese (manganese sulfate) salts. It is packaged in an aluminum pressurized bottle Bag-On-Valve.

### Randomization and blinding

Randomization was stratified into three age groups: 3–12 months, 12–24 months, and 24–48 months. Participants were allocated to the treatment group using three lists of random permuted blocks of size 4 prepared by the statistician using the PLAN procedure of SAS 9.4. Eligible participants were randomly assigned to either the active or control group in order of the randomization scheme prepared by the statistician. In the case of multiple participants from the same family (having the same caretaker in one household), they were placed in the same intervention group. In such a situation, the first child was included in the group according to the randomization list and for the following participant(s), the randomization number was chosen from the list by the investigator. Participants were not blinded to the intervention in this open-labeled study; however, personnel involved with evaluating the participants, as well as the outcome assessors (i.e., statistician) were blinded to the treatment group.

### Outcome assessments

#### Common cold symptoms

Cold symptoms (i.e., nasal congestion, cough, nasal crust, runny nose, thick mucus, and sneezing) were evaluated by caregivers using a questionnaire in the morning and evening throughout the study. Morning evaluations were on the severity of symptoms using a 4-point scale ranging from 0 (not at all) to 3 (severe). Evening evaluations were on the degree of symptom reduction relative to the morning “Since this morning until now, overall, did you observe a reduction in your child’s [symptom]…” using a 4-point scale ranging from 0 (no change) to 3 (substantial improvement). Specific to the active group, additional morning evaluations on Days 0, 1, 2, and 3 were performed within 5, 15, and 30 min after the first product application of the day to assess the short-term effects of the nasal spray.

#### Secondary infection symptoms

Assessment of any secondary infection symptoms related to bronchitis, otitis, tracheitis, pneumonia, or severe fever classified according to an axillary body temperature > 38.0 °C was evaluated daily (evening), from the beginning to the end of the study. Caregivers were asked “Did your child experience any symptoms of infections other than those related to the common cold?” if “Yes” the caregivers checked and ticked all symptoms experienced by their child and rated their severity as mild, moderate, or severe and noted the duration of the symptoms (in hours or “all day”). The subsequent question was “Did you contact your Doctor/study site?” if “Yes” the caregivers noted down what the doctor told them, if “No” they explained why. Symptoms that were listed were common symptoms for bronchitis, otitis, tracheitis, and pneumonia. The marked symptoms were reviewed by the study investigator who then confirmed the secondary infection symptoms. Symptoms that are not commonly related to bronchitis, otitis, tracheitis, and pneumonia, such as gastroenteritis and conjunctivitis were confirmed by the study investigator based on highlighted symptoms in the daily diary.

#### Use of concomitant medication

Recording of concomitant medications (excluding paracetamol) was done by both groups from the beginning to the end of the study. This evaluation took place in the evening, before product application. Caregivers were asked to record the drug name, start date, end date, frequency, and dosage.

#### Use of paracetamol (acetaminophen)

Recording of paracetamol use was done by both groups from the beginning to the end of the study. Paracetamol is generally used for pain, headache or fever relieve. This evaluation took place in the evening, before product application. Caregivers were asked to record the start date, end date, frequency, and dosage.

#### Quality of sleep

Quality of sleep was evaluated by caregivers using a questionnaire every morning from the beginning to the end of the study by both groups. For the active group, the questionnaire was completed before the first product application of the day. The questionnaire contained two questions: “To what extent did your child’s cold symptoms affect your CHILD’S ABILITY TO SLEEP?” and “How much did your child’s cold symptoms affect YOUR OWN ABILITY TO SLEEP?” caregivers were asked to rate each question from a 4-point scale ranging from 0 (not at all) to 3 (severely).

#### Common cold status

Common cold status was evaluated by a concise, two-question global assessment. The first question, “How would you rate your child’s cold symptom relief at this time?” (rated on a 0–3 scale), was completed every morning and evening, before product application from the beginning to the end of the study. Caregivers rated the symptom relief using a 4-point scale ranging from 0 (no relief at all) to 3 (complete relief). The second question, “Do you feel your baby still has a cold? (Yes or No)” was asked to caregivers every morning.

#### Final product survey

At the end of the study, caregivers in the active group completed a product survey inquiring on the overall product satisfaction, the willingness of caregivers to use and recommend the product in the future, ease of use, subject acceptability of product based on the attitude of the child during product administration, and product characteristic (long-lasting effect/fast acting within minutes/lasting moisturization). The assessment of parents/legal guardians’ satisfaction was assessed using a 5-point scale. Product experience was evaluated using a 5-point scale ranging from 1 (disagree completely) to 5 (agree completely).

#### Adverse event assessment

AEs were defined as any untoward medical occurrence or undesirable clinical experience in a participant in a clinical trial, whether or not considered related to the study. This included adverse events resulting from insufficient or inadequate instructions for use, or any malfunction of the nasal spray or from use error or intentional misuse of the nasal spray. AEs were collected via an AE report form, coded using MedDRA dictionary, and categorized as either “not related”, “unlikely”, “possibly”, “probably”, or “definitely” related to the intervention [11]. The severity and causality of all AEs were determined by the study investigator. Information describing the AE collected for assessment and documentation included the following: date of onset/resolution, severity, and causal relationship to study treatment or study conduct (as determined by the study investigator). All documented AEs were followed to resolution or until determined to be clinically insignificant by the study investigator.

### Sample size

No sample size calculation was performed due to insufficient data. Instead, the sample sizes from two clinical studies were referenced [12, 13].

### Statistical analysis

The primary endpoint was the reduction of nasal congestion. Secondary endpoints included reduction of other cold symptoms (nasal crust [dry mucus], runny nose [drip/sniff/snorting/drain-age/discharge], thick mucus, sneezing, coughing), and occurrence of secondary infections (number) daily. Other endpoints included the onset of relief (immediate relief), use of concomitant medications excluding paracetamol, use of paracetamol (frequency), quality of sleep, and global assessment of common cold status. Symptom severity and quality of sleep between groups at each time point were compared using a Mann–Whitney test. Changes from baseline for quality of sleep were evaluated using a Wilcoxon signed-rank test. Persistence of cold symptoms (per the global assessment question) was evaluated using the Chi-square test or Fisher’s exact test when there were < 5 participants answering yes or no at any time point.

Analyses were conducted following a statistical analysis plan developed before the last participant completed the study and before any data were analyzed. Data processing, statistical programming, and analyses were performed using SAS® (SAS Institute, Inc., Cary, NC, USA), version 9.4, and/or Microsoft Excel® 2010 (Microsoft Corp., Redmond, WA). All statistical tests were assessed at the α = 5% level of significance in a bilateral approach. Primary analyses were completed for the Intent-to-Treat (ITT) sample population, which consisted of all enrolled and randomized participants. Additionally, analyses were conducted for the per-protocol (PP) sample population that excluded participants deemed in violation of protocol requirements. Only results for the PP analysis are presented herein, and any meaningful differences between the ITT and PP populations are stated.

## Results

**Fig. 1:**
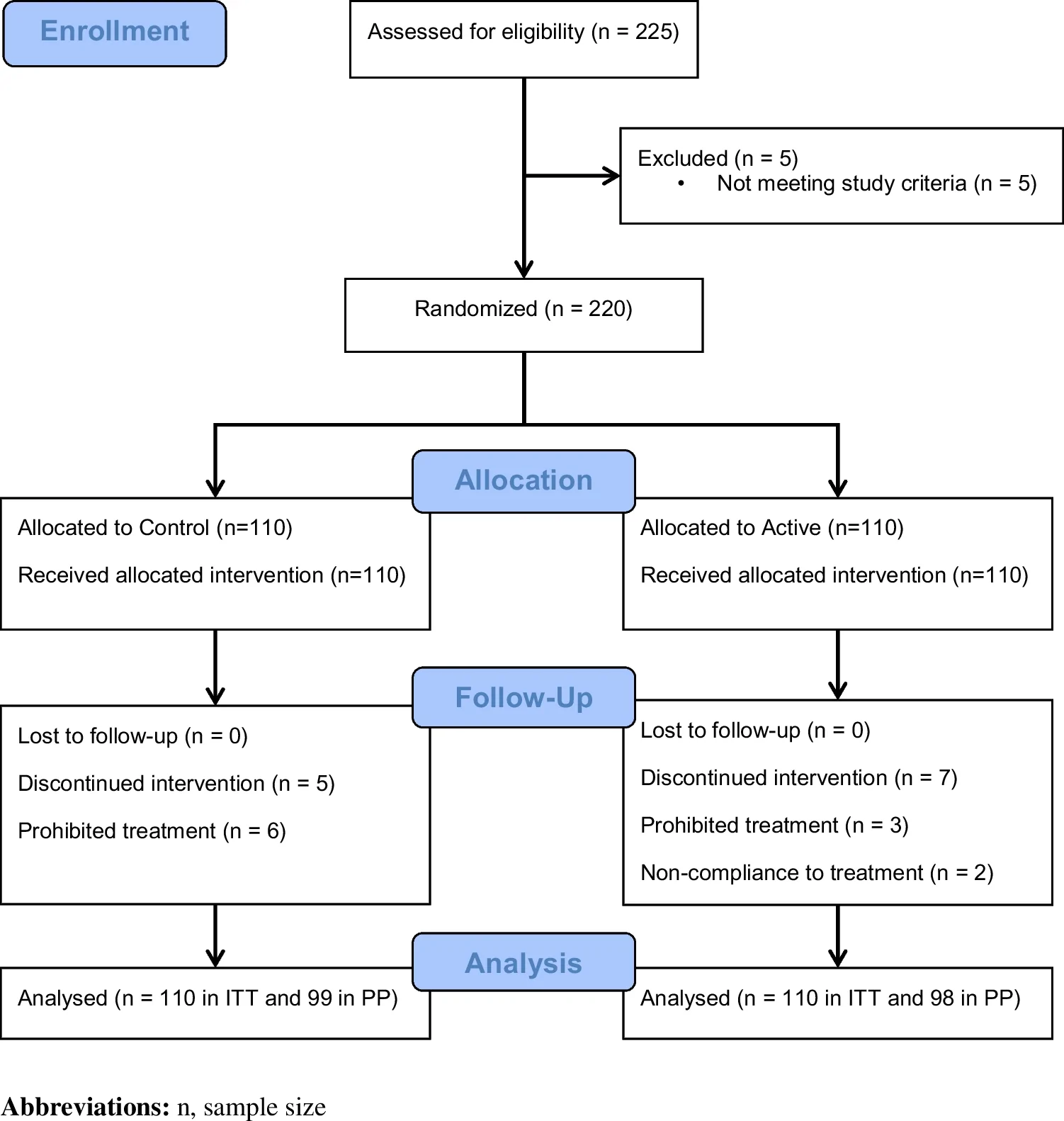
Participant disposition

A total of 225 participants were screened, of whom 5 were excluded for meeting exclusionary criteria Fig. 1. The remaining 220 participants were randomized Fig. 1 to one of two groups: control (standard of care only; *n* = 110) or active (standard of care with nasal spray; *n* = 110). Of the 220 randomized participants, 12 terminated early due to AEs (*n* = 7 in the control group and *n* = 5 in the active group). All 220 randomized participants were included in the ITT population. The PP population (*n* = 197) excluded participants who terminated early as well as violated the study protocol (i.e., non-compliance with nasal spray use, and use of exclusionary products, which are explicitly outlined in the 'Study participants' subsection of the Methods). Baseline characteristics are shown in Table 1. No statistically significant differences in demographic characteristics or baseline symptom severities were observed between the two groups at enrollment.

**Table 1:** Baseline characteristics

	Nasal spray	Control
*n*	95	102
Female (*n*[%])	43 (45.3%)	55 (53.9%)
Male (*n*[%])	52 (54.7%)	47 (46.1%)
Age (min, max)	3–50 months	3–47 months
≤ 12 months (*n*)	27	29
> 12 months (*n*)	68	73

Abbreviations:* n* sample size, max maximum, min minimum.

### Cold symptoms severity

**Fig. 2:**
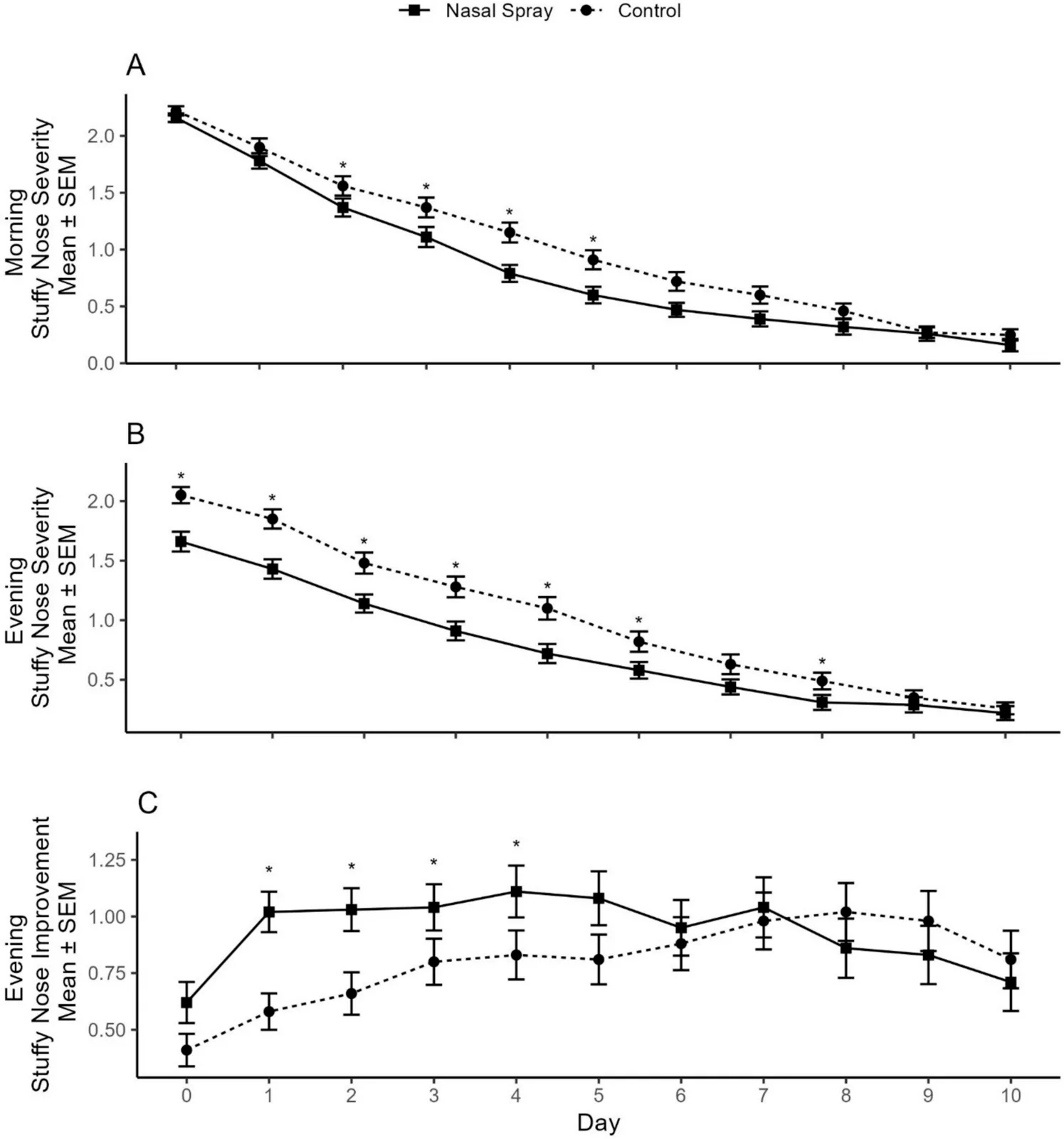
Daily morning (**A**) and evening (**B**) stuffy nose severity score and improvement in stuff nose over time (**C**) in the nasal spray group and control. Between group comparisons at each time point were assessed using a Mann–Whitney test. *P ≤ 0.05 between groups at a specific time point. SEM = standard error of the mean

There was a statistically significant (*p* < 0.05) improvement in the severity of the primary outcome (i.e., nasal congestion) in the active group compared to the control group Fig. 2a and b. The significant results between the groups first appeared on the evening of Day 0, then returned on the evening of Day 1, where they remained significant morning and evening through Day 5 and again on the evening of Day 7. Consistent with this, the subjective rating of improvement in nasal congestion from morning to evening was greater (*p* < 0.05) in the active group compared to the control group through Day 4 Fig. 2c.

**Fig. 3:**
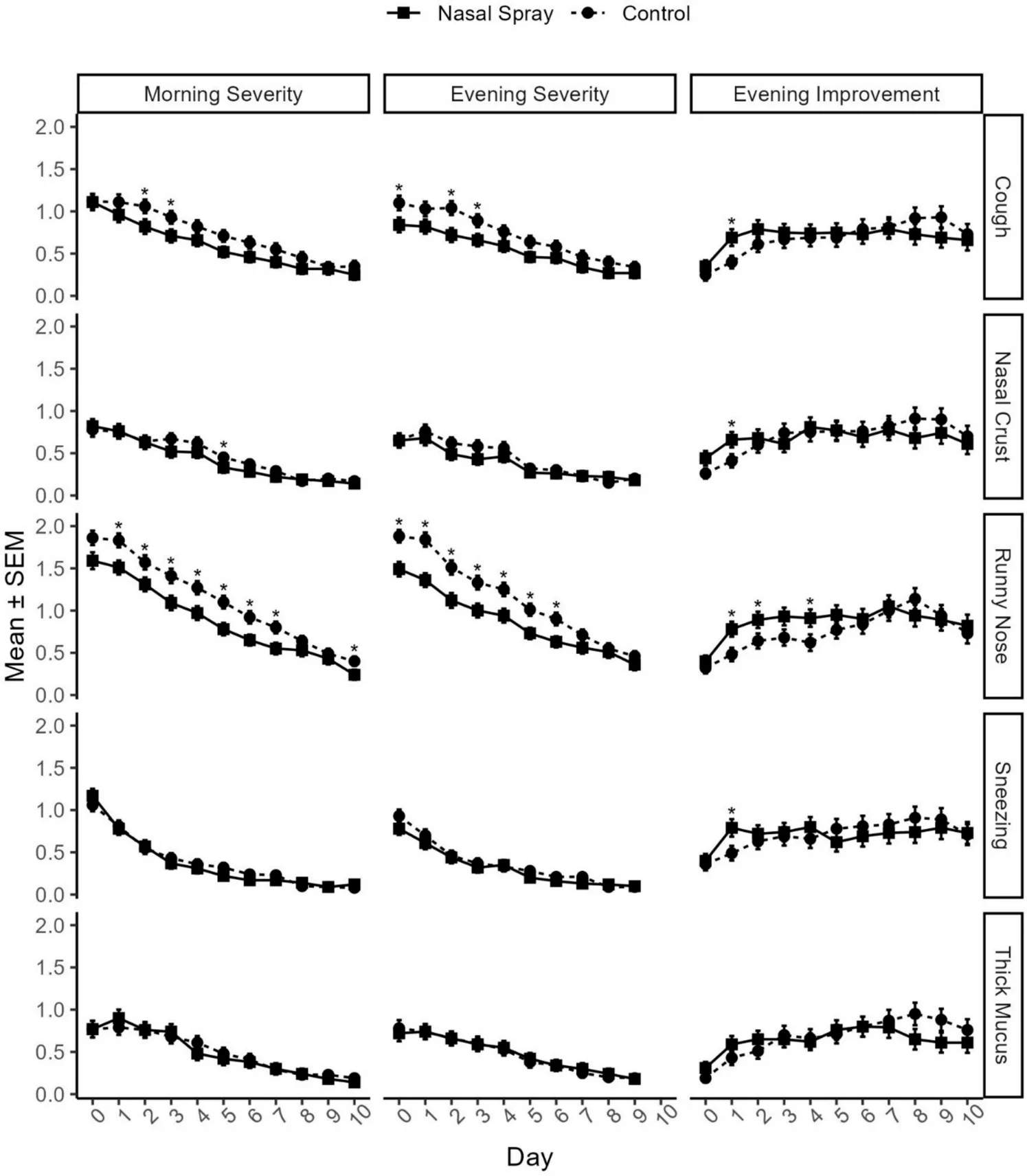
Daily morning and evening severity score and improvement over time from Day 0 to Day 10 in the nasal spray group and control for cough, nasal crust, runny nose, sneezing, and thick mucus. Between group comparisons at each time point were assessed using a Mann–Whitney test. *P ≤ 0.05 between groups at a specific time point. SEM = standard error of the mean

**Fig. 4:**
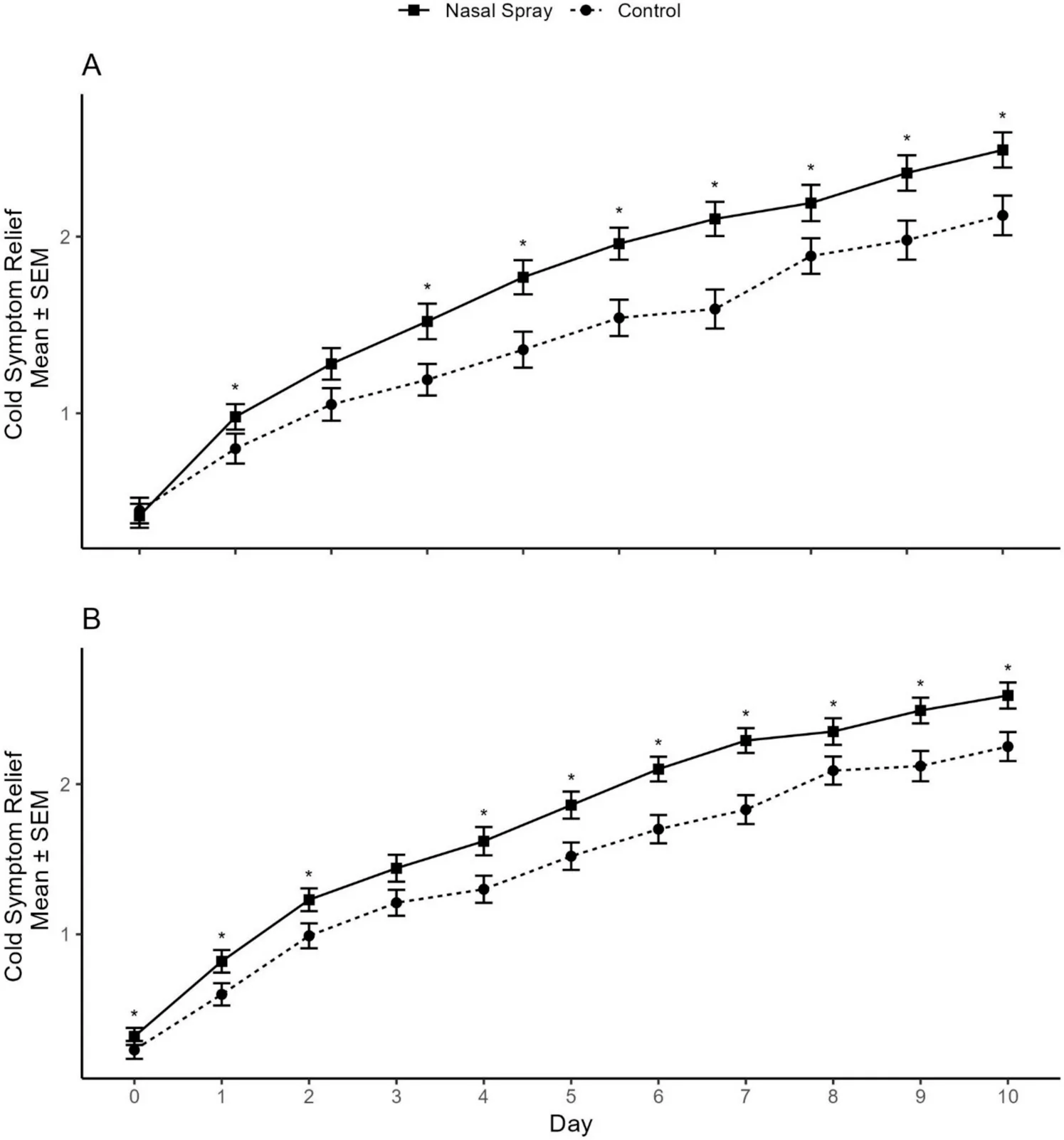
Daily morning (**A**) and evening (**B**) cold symptom relief over time in the nasal spray group and control. Between group comparisons at each time point were assessed using a Mann–Whitney test. *P ≤ 0.05 between groups at a specific time point. SEM = standard error of the mean

The addition of nasal spray use consistently decreased (*p* < 0.05) the severity of coughs and runny nose Fig. 3. In the long-term daily evaluations over the 10-day period, little, if any, differences between groups were observed for morning and evening nasal crust, thick mucus, and sneezing severity, and this may be due to the overall low occurrences of these symptoms in either group Fig. 3. Overall subjective rating of cold symptom relief was better (*p* < 0.05) in the active group compared to the control group Fig. 4. Particularly, morning relief scores were significantly larger with the Sterimar nasal spray use than the standard of care (*p* < 0.05), suggesting a long-lasting effect of the spray on nasal symptom relief. In terms of individual symptoms, subjective rating of improvement was most consistent for runny nose, which was better (*p* < 0.05) in the active group compared to the control group; other symptoms showed little to no differences between groups Fig. 3.

The decrease in nasal congestion was shown to be immediate based on significant (*p* < 0.05) improvements compared to baseline within 5, 10, and 15 min of nasal spray use Table 2. However, when evaluating the effects specifically at 5, 15, and 30 min after the first product application on Days 0 to 3, immediate relief from coughs, nasal crust, runny nose, thick mucus, and sneezing was evident (*p* < 0.05) for the active group Table 2.

**Table 2:** Severity of cold symptoms before use of nasal spray and change from baseline at 5, 15, and 30 min after use of nasal spray on Day 0 to Day 3

Day 0				
Symptoms	Before use	t5	t15	t30
Nasal congestion	2.16 (0.37)	−0.84 (0.80)*	−1.03 (0.87)*	−1.17 (0.91)*
Cough	1.11 (0.93)	−0.40 (0.76)*	−0.45 (0.78)*	−0.47 (0.82)*
Nasal crust	0.82 (0.82)	−0.40 (0.80)*	−0.43 (0.90)*	−0.48 (0.86)*
Runny nose	1.59 (0.97)	−0.21 (0.99)*	−0.37 (1.05)*	−0.52 (1.06)*
Thick mucus	0.77 (0.97)	−0.21 (0.73)*	−0.20 (0.79)*	−0.25 (0.77)*
Sneezing	1.17 (0.80)	−0.54 (0.84)*	−0.69 (0.88)*	−0.73 (0.92)*
Day 1				
Symptoms	Before use	t5	t15	t30
Nasal congestion	1.78 (0.66)	−0.43 (0.72)*	−0.73 (0.82)*	−0.88 (0.86)*
Cough	0.96 (0.86)	−0.28 (0.68)*	−0.40 (0.71)*	−0.43 (0.71)*
Nasal crust	0.76 (0.85)	−0.34 (0.66)*	−0.43 (0.74)*	−0.47 (0.70)*
Runny nose	1.51 (0.81)	−0.23 (0.54)*	−0.40 (0.71)*	−0.48 (0.72)*
Thick mucus	0.90 (0.98)	−0.25 (0.62)*	−0.26 (0.64)*	−0.31 (0.72)*
Sneezing	0.78 (0.75)	−0.21 (0.57)*	−0.22 (0.62)*	−0.31 (0.65)*
Day 2				
Symptoms	Before use	t5	t15	t30
Nasal congestion	1.37 (0.77)	−0.37 (0.62)*	−0.60 (0.61)*	−0.66 (0.65)*
Cough	0.82 (0.85)	−0.13 (0.62)*	−0.22 (0.61)*	−0.24 (0.66)*
Nasal crust	0.63 (0.79)	−0.27 (0.63)*	−0.32 (0.62)*	−0.34 (0.65)*
Runny nose	1.31 (0.80)	−0.14 (0.56)*	−0.24 (0.61)*	−0.31 (0.64)*
Thick mucus	0.76 (0.92)	−0.11 (0.52)	−0.19 (0.53)*	−0.22 (0.60)*
Sneezing	0.57 (0.72)	−0.12 (0.48)*	−0.16 (0.51)*	−0.18 (0.58)*
Day 3				
Symptoms	Before use	t5	t15	t30
Nasal congestion	1.11 (0.86)	−0.25 (0.55)*	−0.44 (0.65)*	−0.53 (0.73)*
Cough	0.71 (0.76)	−0.09 (0.55)	−0.16 (0.59)*	−0.18 (0.62)*
Nasal crust	0.52 (0.71)	−0.21 (0.51)*	−0.25 (0.52)*	−0.26 (0.57)*
Runny nose	1.09 (0.84)	−0.09 (0.49)	−0.17 (0.58)*	−0.23 (0.57)*
Thick mucus	0.74 (0.87)	−0.15 (0.55)*	−0.21 (0.62)*	−0.23 (0.66)*
Sneezing	0.37 (0.62)	−0.05 (0.30)	−0.08 (0.43)	−0.12 (0.38)*

t5, t15, and t30 correspond to 5, 15, and 30 min post nasal spray use.

*P ≤ 0.05 between groups at a specific time point.

### Cold recovery

**Fig. 5:**
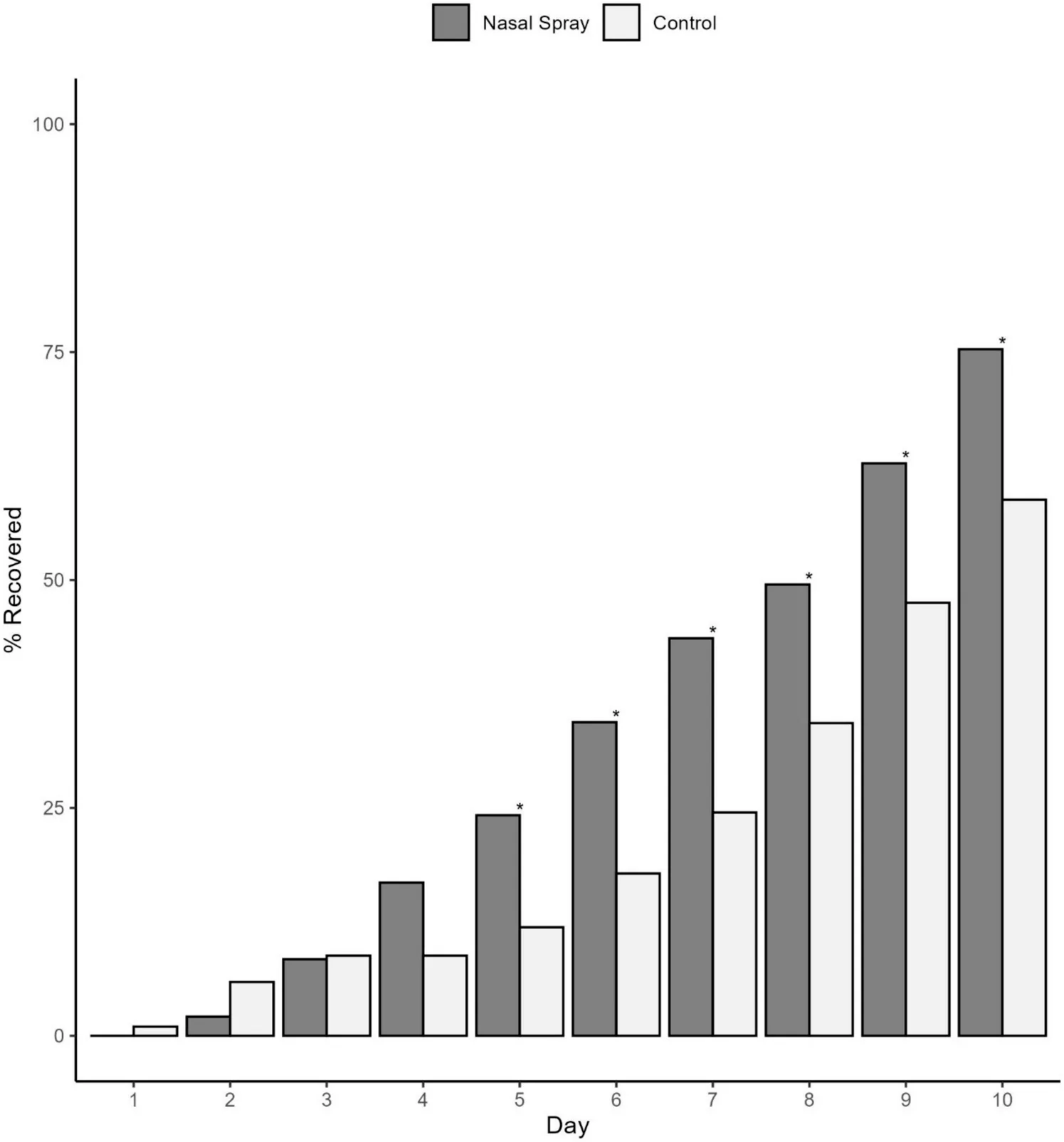
Percentage of children who recovered from cold in the morning of the respective days. Between-group comparisons at each time point were assessed using a Chi-square test. *P ≤ 0.05 between groups at a specific time point

During the first 4 days, the proportion of recovered children was not different between the 2 groups. From Day 5 onwards, the difference was statistically significant (*p* < 0.05), with a larger proportion of recovered children in the active group, reaching 75.3% vs. 58.5% on Day 10 Fig. 5.

### Quality of sleep

The quality of the child’s sleep and the caretaker’s ability to sleep were minimally affected by the nasal spray (Supplemental Fig. 1).

### Secondary infection symptoms

There were no significant differences in the proportion of participants experiencing secondary infection symptoms (fever, ear infection, pneumonia, bronchitis, severe cough, sore throat) between the groups (Supplemental Fig. 2). In addition, participants treated with the study product presented secondary infection symptoms, such as diarrhea and wheezing, whereas participants treated with the standard of care presented fluid draining from the ear(s) as an additional secondary infection symptom. Most of those symptoms lasted 1–2 days. Secondary infection symptoms reported include appetite loss, back and muscle pain, body discomfort/not feeling well, breathing difficulty, cough, ear pain, fever, irritability, noisy breathing sound, sleeping difficulty, sore throat, tiredness (fatigue), and vomiting or gagging.

### Use of concomitant medications

26 participants in the active group and 17 participants in the control group took concomitant medication. Per the study’s exclusion criteria, these permitted medications did not include treatments that could confound the results, such as other topical nasal therapies, decongestants, antihistamines, or antibiotics. The number of participants who used paracetamol was comparable between groups (5 in the active group and 4 in the control group).

### Product survey

93.6% of the caregivers liked the product, and 89.4% were satisfied with how well the product worked overall. 92.6% of the responders would use this product for their child in the future (Supplemental Table 1). 88.2% of the responders agreed that the type of spray bottle is the most effective to unblock their child’s nose, 81.7% agreed that the product provides long-lasting relief, and 89.4% agreed that the product is fast-acting within minutes.

### Adverse events

246 adverse events were reported in 106 participants during the investigation. None of the adverse events were related to the nasal spray device. No serious adverse event occurred during the study.

## Discussion

This study demonstrated that the addition of a hypertonic seawater-based nasal spray (Stérimar Blocked Nose Baby) to standard care provided significant immediate relief and faster resolution of nasal congestion and other cold symptoms in infants and toddlers with the common cold compared to standard care alone. Additionally, using a hypertonic nasal spray helped recovery from a cold sooner than standard care. This is among the first studies to evaluate the safety, efficacy, and tolerability of a non-pharmacologic nasal spray in this vulnerable age group, addressing an important gap in pediatric respiratory care.

Our findings are consistent with prior studies demonstrating the efficacy of nasal irrigation in alleviating common cold symptoms, including cough and runny nose [14]. Additionally, a recent systematic review affirms that saline nasal irrigation is a safe and effective adjunctive treatment for pediatric URTIs, significantly improving nasal congestion and sleep quality with minimal adverse effects [15]. While most research on nasal irrigation in children has focused on those with underlying conditions (e.g., chronic sinusitis, allergic rhinitis, chronic tonsillitis, bronchiolitis), limited data exist for otherwise healthy children experiencing acute colds or influenza [8, 16, 17]. For instance, a 12-week multicenter, randomized trial involving 401 children aged 6–10 years with uncomplicated colds or flu found that adding isotonic saline irrigation to standard medication (e.g., antipyretics, nasal decongestants, mucolytics) significantly reduced nasal secretion severity, nasal obstruction, sore throat, and medication use within three weeks compared to standard care alone [8]. Similarly, a randomized, placebo-controlled trial of 69 children (3–12 years) demonstrated that isotonic saline irrigation improved rhinorrhea, nasal congestion, cough, sleep quality, and Rhinoconjunctivitis Quality of Life Questionnaire scores [17]. Another study, which targeted younger children aged 2–17 months with acute upper respiratory infections, compared isotonic saline drops, hypertonic seawater drops, and no treatment. Both nasal irrigation groups showed reduced symptom severity (e.g., congestion, rhinorrhea, cough) and improved sleep quality after one week, with no significant differences between the saline types [16]. These collective findings corroborate the efficacy of nasal irrigation in reducing symptoms across healthy pediatric populations (3–48 months), reinforcing the relevance of our results.

Additionally, unlike these previous studies, we also investigated the efficacy of our product on immediate relief. Cold symptom relief was not only evident the evening of the first day of use but statistically significant improvements in nasal congestion, cough, nasal crust, runny nose, thick mucus, and sneezing were observed within 5 and 15 min of use, underscoring its fast-acting nature. Such rapid symptom relief is critical in managing cold-related discomfort, particularly in young children, and aligns with caregiver reports, whereby 89.4% of them agreed that the product was fast-acting. Indeed, the popularity of nasal decongestants is partly due to their fast-acting nature [18]. The ability to alleviate symptoms rapidly may contribute to enhanced comfort for individuals, particularly children, during the acute phase of illness, potentially improving the overall experience of care and reducing the burden of the cold on both patients and caregivers. Additionally, the product survey results support the long-lasting symptom relief effect of Sterimar nasal spray. This effect is further validated by the finding that there was no rebound in symptom severity between the mornings and the previous evenings. Typically, nasal symptoms are expected to worsen overnight when the product cannot be used. In support, caregivers of children in the active group overwhelmingly endorsed the nasal spray and expressed a strong willingness to use the product again for their child. A prior survey of caregivers whose children were prescribed nasal saline irrigation revealed a significant disconnect between expectations and reality: while only 28% of parents initially believed their children would tolerate nasal saline irrigation, 86% of children were reported to tolerate it successfully [19]. This highlights a key barrier to its routine recommendation—misperceptions by both parents and physicians regarding children’s tolerance for the intervention. Thus, our findings have practical implications, as they contribute to improving parental acceptance of nasal saline irrigation as a non-pharmacological alternative for symptom relief.

Both isotonic saline and hypertonic seawater nasal solutions likely alleviated nasal congestion through the mechanical cleansing of the nasal passages, effectively flushing out mucus, allergens, and pathogens. By reducing nasal congestion, which contributes to post-nasal drip, these solutions may have indirectly alleviated associated symptoms such as coughing and runny nose, often exacerbated by mucus drainage. While no significant differences were observed for daily morning and evening symptom scores of nasal crust, thick mucus, or sneezing, this may reflect the low prevalence of these symptoms within the study population. Importantly, the minimal impact on these less common symptoms does not diminish the overall effectiveness of the nasal spray, which demonstrated a clear benefit in relieving nasal congestion and related discomfort. Hypertonic solutions, such as seawater-based formulations, may provide additional therapeutic advantages over isotonic saline. By creating an osmotic gradient, hypertonic solutions can draw water from swollen mucosal tissues into the nasal lumen, potentially reducing edema, improving mucociliary clearance, and decreasing nasal resistance [14]. Furthermore, hypertonic solutions may enhance mucociliary function by maintaining hydration and facilitating the clearance of debris and inflammatory mediators [14]. The inclusion of trace elements like manganese and copper in the hypertonic seawater formulation may also contribute to its therapeutic effects. Manganese supports antioxidant defenses through superoxide dismutase activity, while copper plays a role in tissue repair and immune modulation [14]. The literature on nasal irrigation in populations with diverse conditions (e.g., allergic rhinitis, upper respiratory tract infections [URTIs], chronic sinusitis) has shown mixed results regarding the superiority of hypertonic versus isotonic solutions. Some studies report no differences between the two, while others suggest greater benefits with hypertonic solutions [14]. In children with acute URTIs, recent systematic evidence demonstrates that while both formulations yield symptom improvement, hypertonic saline may offer marginally superior outcomes, particularly in addressing nasal obstruction [15].

Secondary outcomes, such as sleep quality and secondary infection rates, showed no significant differences between groups. These results may reflect an underpowered study design for detecting subtle differences in secondary outcomes. Future research could use objective tools such as actigraphy or polysomnography to better evaluate sleep patterns and disturbances caused by congestion. Similarly, assessing secondary infections may benefit from considering viral types, immune responses, and environmental factors. Importantly, while specific secondary infection symptoms such as diarrhea and wheezing were reported in the active group, these represent the natural progression of the underlying viral illness rather than adverse effects of the hypertonic seawater solution, consistent with the investigator's determination that no adverse events were causally related to the device. Another limitation of this study is its open-label design, which may introduce bias in reporting outcomes like symptom severity and sleep quality, as caregivers may perceive the active treatment as more effective. However, a blinded study design is not feasible for testing the effect of using a nasal spray plus standard care compared to standard care alone. Additionally, the short duration of the intervention (up to 10 days) limits the ability to assess long-term effects, including the occurrence of secondary infections. Objective measures of nasal congestion, such as rhinomanometry, were not utilized, which might have provided additional validation of the findings. While some participants required permitted concomitant medications, our strict exclusion criteria ensured no confounding respiratory treatments were used. Furthermore, any remaining potential for confounding from these permitted medications was mitigated by the randomized controlled study design, which naturally distributed unmeasured variables evenly between the study arms.

## Conclusions

This study demonstrates that the addition of a hypertonic seawater-based nasal spray to standard care provides significant and immediate relief from nasal congestion and other cold symptoms in infants and toddlers. As one of the few studies to evaluate non-pharmacologic nasal sprays in this vulnerable population, it highlights the spray’s rapid onset of action, safety, and high caregiver satisfaction, making it a valuable, well-tolerated option for pediatric cold symptom management. These findings reinforce the efficacy of nasal irrigation in reducing the burden of common cold symptoms, addressing an important gap in pediatric care. Future research should explore its applications in recurrent URTIs, allergic rhinitis, and other respiratory conditions to fully elucidate its role in improving pediatric respiratory health.

## Supplementary Information

Below is the link to the electronic supplementary material.

Supplementary file1 (PDF 195 KB)

Supplementary file2 (PDF 102 KB)

Supplementary file3 (PDF 315 KB)

## Data Availability

The datasets generated during and/or analyzed during the current study are available from the corresponding author on request.

## Abbreviations

AE(s): Adverse event(s)
CONSORT: Consolidated standards of reporting trials
EN ISO: European standard and international standard
EU: European Union
ITT: Intent-to-treat
*n*: Sample size
OTC: Over the counter
PP: Per protocol
RADT: Rapid antigen detection test
RSV: Respiratory syncytial virus
SAS: Statistical analysis system
URTI(s): Upper respiratory tract infection(s)
