# Supplementary material for: Efficacy and safety of seawater-based nasal irrigation on nasal congestion in infants and toddlers with common cold: a randomized controlled trial
Source: Eur Arch Otorhinolaryngol. 2026 Sep 16;283(12):7945–57. doi: 10.1007/s00405-026-10615-z (PMC13768243; doi:10.1007/s00405-026-10615-z)

**Online Resource 1. Ability to sleep ratings for child (A) and caretaker (B).** Between group comparisons at each time point were assessed using a Mann–Whitney test. *P≤0.05 between groups at a specific time point. SEM = standard error of the mean.

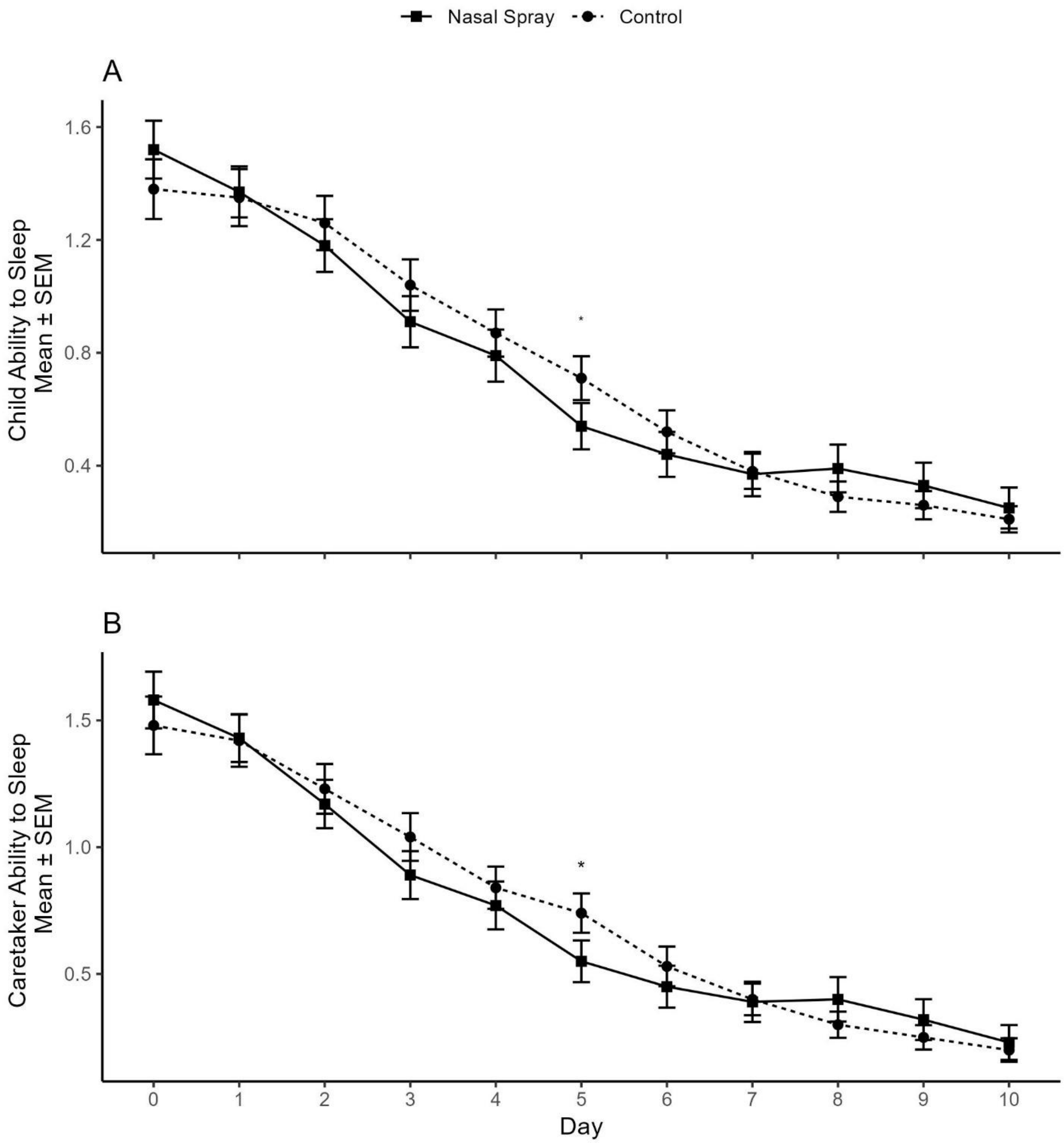

Supplement: Supplementary file 1 [file 405_2026_10615_MOESM1_ESM.pdf]
