# Supplementary material for: Efficacy and safety of seawater-based nasal irrigation on nasal congestion in infants and toddlers with common cold: a randomized controlled trial
Source: Eur Arch Otorhinolaryngol. 2026 Sep 16;283(12):7945–57. doi: 10.1007/s00405-026-10615-z (PMC13768243; doi:10.1007/s00405-026-10615-z)

Online Resource 2. Presence of secondary infection symptoms in the nasal spray and control groups. Comparison between groups was not performed.

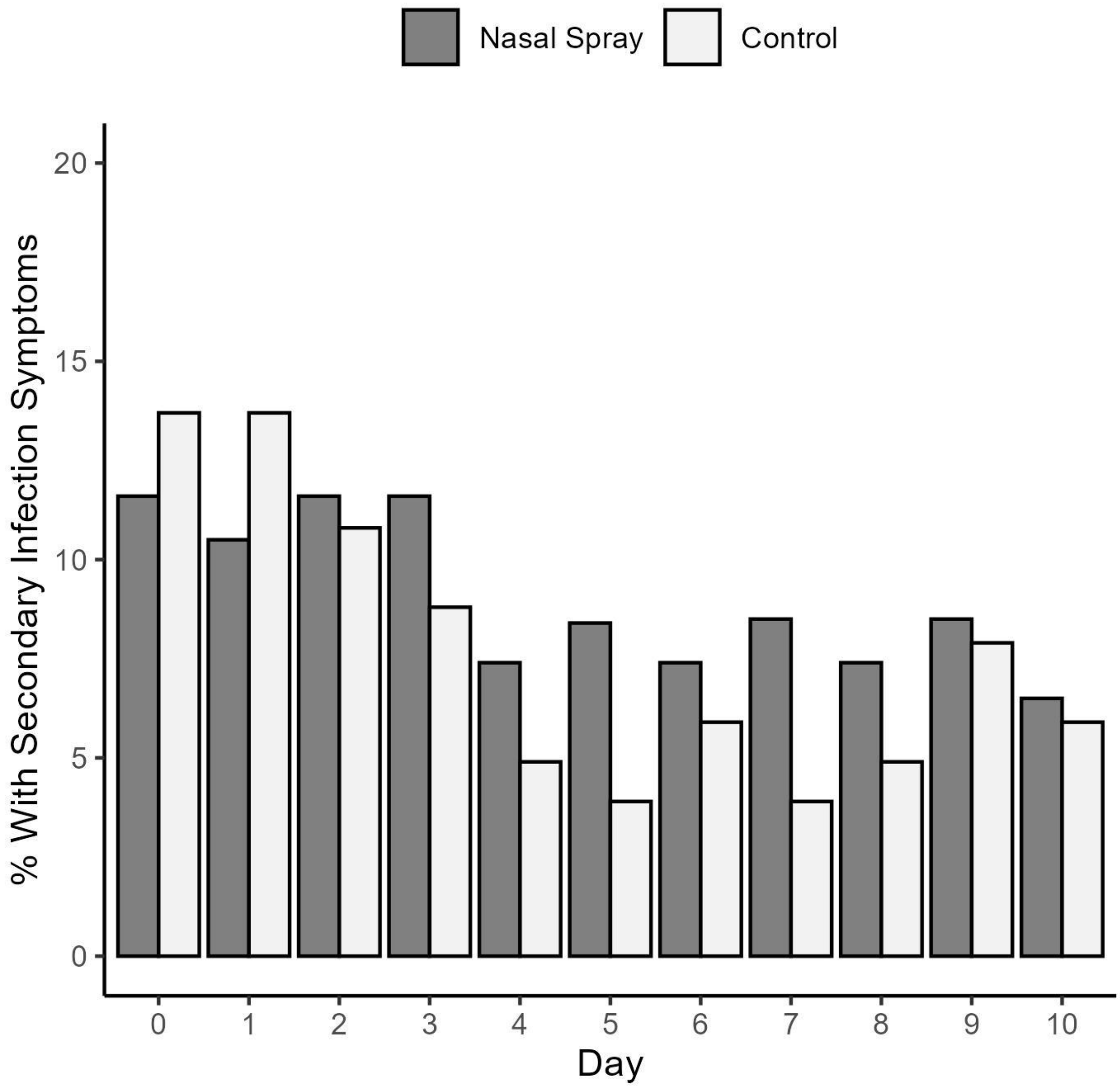

Supplement: Supplementary file 2 [file 405_2026_10615_MOESM2_ESM.pdf]
