# Supplementary material for: Efficacy and safety of seawater-based nasal irrigation on nasal congestion in infants and toddlers with common cold: a randomized controlled trial
Source: Eur Arch Otorhinolaryngol. 2026 Sep 16;283(12):7945–57. doi: 10.1007/s00405-026-10615-z (PMC13768243; doi:10.1007/s00405-026-10615-z)
Supplement: Supplementary file 3 [file 405_2026_10615_MOESM3_ESM.pdf]

Online Resource 3. Responses for product survey for the nasal spray group only. Only descriptive analysis was performed.

		N (%)	CI 95%
How much do you like or dislike this product?	N (MV)	94 (1)	
	Like	88 (93.6%)	[86.8% ,97.0%]
	Dislike	6 (6.4%)	[3.0% ,13.2%]
How much do you like or dislike this spray bottle?	N (MV)	94 (1)	
	Like	89 (94.7%)	[88.1% ,97.7%]
	Dislike	5 (5.3%)	[2.3% ,11.9%]
How did your child respond to the product application?	N (MV)	93 (2)	
	Satisfied	60 (64.5%)	[54.4% ,73.5%]
	Dissatisfied	33 (35.5%)	[26.5% ,45.6%]
How satisfied are you with how well the product worked overall?	N (MV)	94 (1)	
	Satisfied	84 (89.4%)	[81.5% ,94.1%]
	Dissatisfied	10 (10.6%)	[5.9% ,18.5%]
How likely would you be to use this product for your child in the future?	N (MV)	94 (1)	
	Would use	87 (92.6%)	[85.4% ,96.3%]
	Would not use	7 (7.4%)	[3.7% ,14.6%]
How likely would you be to recommend this product to a friend?	N (MV)	94 (1)	
	Likely	80 (85.1%)	[76.5% ,90.9%]
	Not likely	14 (14.9%)	[9.1% ,23.5%]
The product is easy to use	N (MV)	93 (2)	
	Agree	93 (100.0%)	[96.0% ,100.0%]
	Disagree	0 (0.0%)	
The product is easy to clean	N (MV)	94 (1)	
	Agree	93 (98.9%)	[94.2% ,99.8%]
	Disagree	1 (1.1%)	[0.2% ,5.8%]

		N (%)	CI 95%
The product easy to carry with me during the day	N (MV)	93 (2)	
	Agree	80 (86.0%)	[77.5% ,91.6%]
	Disagree	13 (14.0%)	[8.4% ,22.5%]
The strength of the spray is appropriate for my baby	N (MV)	94 (1)	
	Agree	87 (92.6%)	[85.4% ,96.3%]
	Disagree	7 (7.4%)	[3.7% ,14.6%]
The type of spray bottle is the most effective to unblock my baby’s nose	N (MV)	93 (2)	
	Agree	82 (88.2%)	[80.1% ,93.3%]
	Disagree	11 (11.8%)	[6.7% ,19.9%]
I would not use this product without healthcare professional advice due to its type of spray bottle	N (MV)	93 (2)	
	Agree	20 (21.5%)	[14.4% ,30.9%]
	Disagree	73 (78.5%)	[69.1% ,85.6%]
The product is intuitive to use	N (MV)	93 (2)	
	Agree	90 (96.8%)	[90.9% ,98.9%]
	Disagree	3 (3.2%)	[1.1% ,9.1%]
I feel confident using this product on my baby	N (MV)	94 (1)	
	Agree	90 (95.7%)	[89.6% ,98.3%]
	Disagree	4 (4.3%)	[1.7% ,10.4%]
The product provides long-lasting relief	N (MV)	93 (2)	
	Agree	76 (81.7%)	[72.7% ,88.3%]
	Disagree	17 (18.3%)	[11.7% ,27.3%]
The product provides long-lasting decongestion	N (MV)	93 (2)	
	Agree	73 (78.5%)	[69.1% ,85.6%]
	Disagree	20 (21.5%)	[14.4% ,30.9%]
The product is fast-acting within minutes	N (MV)	94 (1)	
	Agree	84 (89.4%)	[81.5% ,94.1%]
	Disagree	10 (10.6%)	[5.9% ,18.5%]

		N (%)	CI 95%
The product provides lasting comfort to my baby	N (MV)	94 (1)	
	Agree	70 (74.5%)	[64.8% ,82.2%]
	Disagree	24 (25.5%)	[17.8% ,35.2%]
The product is effective throughout the day	N (MV)	94 (1)	
	Agree	65 (69.1%)	[59.2% ,77.6%]
	Disagree	29 (30.9%)	[22.4% ,40.8%]
The product is effective throughout the night	N (MV)	94 (1)	
	Agree	68 (72.3%)	[62.6% ,80.4%]
	Disagree	26 (27.7%)	[19.6% ,37.4%]

Abbreviations: MV, missing value; N, sample size
